# Ecologically informed solar enables a sustainable energy transition in US croplands

**DOI:** 10.1073/pnas.2501605122

**Published:** 2025-04-21

**Authors:** Matthew A. Sturchio, Adam Gallaher, Steven M. Grodsky

**Affiliations:** ^a^Department of Natural Resources and the Environment, Cornell University, Ithaca, NY 14853; ^b^Department of Biology, Colorado State University, Fort Collins, CO 80523; ^c^US Geological Survey, New York Cooperative Fish and Wildlife Research Unit, Department of Natural Resources and the Environment, Cornell University, Ithaca, NY 14853

**Keywords:** ecosystem services, solar energy, sustainable agroecosystems, corn ethanol

## Abstract

United States (US) croplands are ideal recipient environments for solar photovoltaic (PV) energy because they are flat and have a high solar resource. Perceived threats of solar to agriculture have led some stakeholders to suggest that croplands be exclusively used to produce food. However, 12 million hectares of US croplands, an area about the size of New York State, are already dedicated to corn grown for ethanol (i.e., biofuel), an energy product that requires significantly more land than solar PV per unit energy. Ecosystem service benefits of an ecologically informed approach to solar development (i.e., ecovoltaics), coupled with significant land-use advantages over corn ethanol, make solar an attractive solution for a sustainable energy transition in croplands. Here, we evaluated how the conversion of a small fraction of corn-ethanol croplands into ecovoltaic solar facilities might improve land-use efficiency of energy generation, enhance ecosystem services, and provide landscape diversification. Through spatial analyses, we determined that converting just 3.2% of land currently used for corn ethanol would increase the share of utility-scale solar energy in the US from 3.9 to 13%. We also identified target locations where strategic conversion of corn ethanol to solar PV colocated with perennial vegetation could filter excess nutrients transported from adjacent farm runoff, diversify and connect agricultural landscapes, and provide local wildlife habitat. In contrast to the common perception of land-use competition and land scarcity in the energy transition, our findings highlight benefits of colocated energy landscapes that integrate fundamental principles of energy development and sustainable agroecosystems.

## Significance

Solar energy is often framed as a threat to croplands. However, vast croplands in the midwestern United States already support corn ethanol biofuel, a form of energy with a greater land-use footprint per unit energy (~30×) and potential environmental impact (e.g., excessive fertilizer application) than solar energy. We envisioned sustainable agroecosystems that can result from converting a small proportion of corn ethanol croplands into ecologically informed solar facilities (i.e., ecovoltaics). We identified opportunities where the strategic placement of ecovoltaic solar could facilitate enhancement of ecosystem services like water quality, pollination services, and wildlife habitat in crop-dominated ecosystems. Our analysis offers an ecologically informed solution to facilitate a sustainable energy transition in croplands via colocated solar energy and ecosystem services.

Expansion of solar photovoltaic (PV) energy generation in rural communities of the United States (US) has sparked concern regarding displacement of highly productive croplands (1–5). At face value, utilization of croplands for energy infrastructure contradicts United Nations Sustainable Development Goals (6), namely zero hunger (e.g., food security) and economic stability (e.g., loss of agricultural jobs). Indeed, negative environmental consequences associated with land used solely for PV, especially in sensitive natural ecosystems, have been well established (7–12). However, alternative approaches to solar development that colocate agriculture and PV energy (i.e., agrivoltaics) or intentionally coprioritize PV energy and ecosystem services (i.e., ecovoltaics; see ref. 13) have been proposed as potential solutions for alleviating land-use tension through a combination of economic and environmental benefits (13–19).

**Fig. 1.**
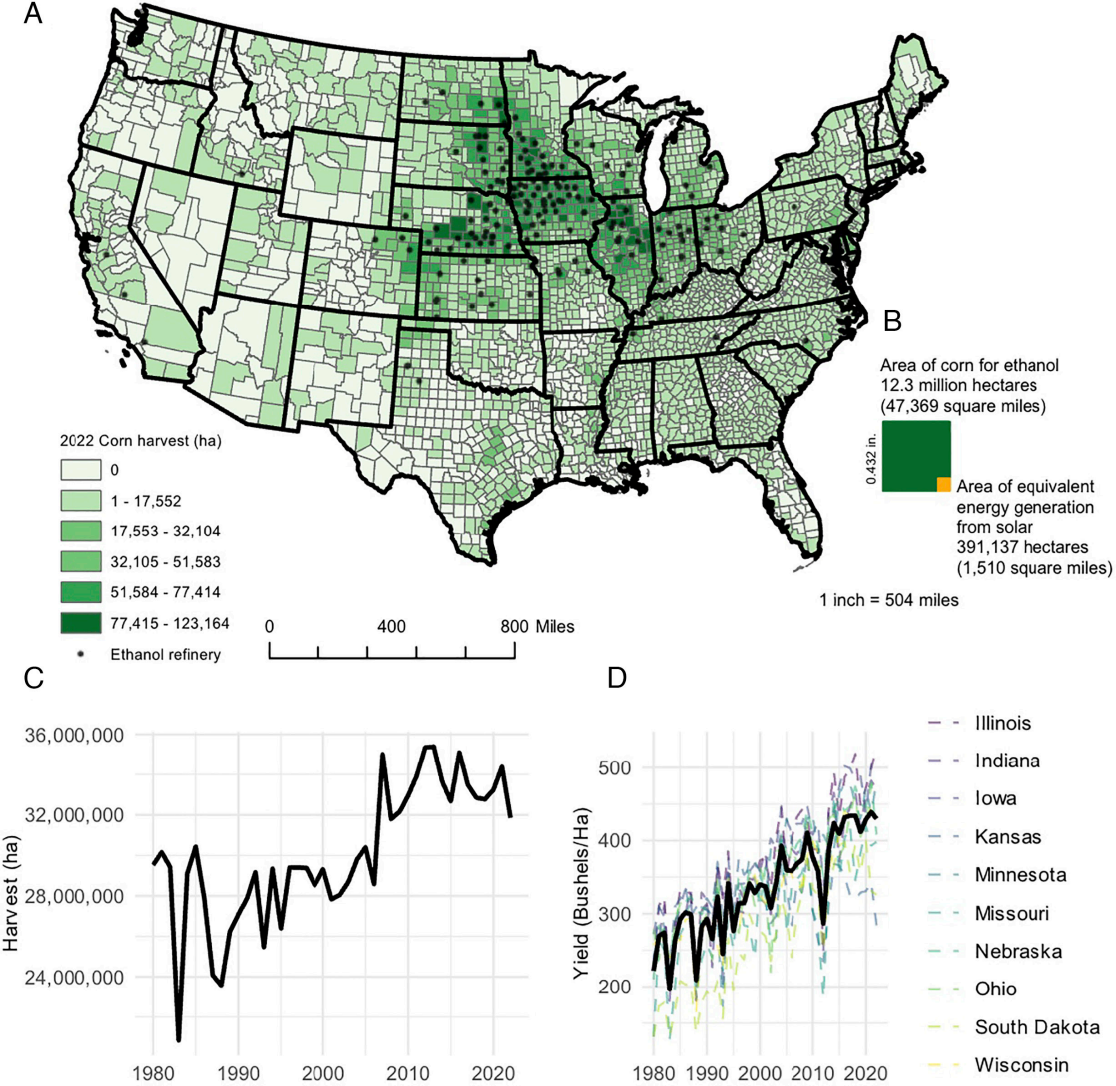
(*A*) Spatial overlap of corn harvest and ethanol refineries in 2022 (28, 29). (*B*) visual representation of land area required for equivalent energy content of corn ethanol from utility-scale solar energy (*SI Appendix, Supplemental Methods*). (*C*) Total area of corn grain harvest from 1980 to 2022 (29) and (*D*) corn grain yield (BU/ha) of top 10 corn producing states from 1980 to 2022 (29); the black line represents average yield (BU/ha) over time. Note that corn-ethanol land use (*B*) represents 30 to 40% of total corn land use (*A*) in the contiguous US.

Results from foundational studies currently support ecovoltaic principles at scale in the midwestern US (20, 21). Successful establishment of native, perennial vegetation in solar arrays (*SI Appendix*, S1) with subsequent visitation and use by pollinators, indicate opportunities for the strategic deployment of PV arrays that provide ecosystem-service enhancement in highly homogenized croplands (13, 20, 21). However, social opposition to solar development in croplands persists, and moratoriums on solar development are underpinned by the argument that prime agricultural land should be left to produce food (3, 22, 23). Meanwhile, approximately 12 million hectares (ha) of croplands (an area about the size of New York State), are cultivated for energy production in the form of corn ethanol (Fig. 1*A*; 24), a vast majority of which are concentrated in the midwestern US (e.g., ~93%; Fig. 1*A*; 25). Passage of the Clean Air Act amendments in 1990, banning a common gasoline additive (i.e., methyl tert-buryl ether) and passage of federal biofuel energy policies (i.e., Energy Policy Act 2005 and Energy Independence and Security Act 2007), led to a rapid expansion of ethanol production and consumption (*SI Appendix*, S2; 26). Ethanol production in 2022 totaled 15.3 billion gallons, up from 83 million gallons in 1981 (27). Furthermore, corn designated to ethanol production in 2022 represented a 231% increase from 2005 levels (*SI Appendix*, S3; 24).

Expansion of corn land use (Fig. 1*C*) has contributed to conversion of native prairie ecosystems and homogenization of crop landscapes (30–33); such reductions in native vegetation subsequently deplete biodiversity and detrimentally impact ecosystem services upon which humans rely (e.g., water quality, soil stabilization, and carbon sequestration; 33–35). Conventional management strategies associated with corn production (e.g., soil tillage, excessive fertilizer and water use, and intensive use of pesticides) are known to negatively affect natural resources as well as directly and indirectly alter ecosystem processes (36–43). With respect to greenhouse gas (GHG) emissions, it remains unclear whether corn ethanol is a realistic solution for achieving a net-zero future; optimistic estimates only found a 43% reduction in GHG emissions when comparing corn ethanol to gasoline (44), while analyses more critical of the effect that land-use change to corn has on carbon sequestration suggest that corn ethanol could be 24% *more* carbon-intensive than gasoline (45, 46). Corn also requires significant amounts of water and fertilizer, both of which are emissions intensive in their own right (e.g., pumping for irrigation and the Haber–Bosch process for synthetic nitrogen; 47–49). Aside from GHG emissions, intensive agriculture contributes to soil erosion, reduces water quality, and homogenizes landscapes, thus increasing vulnerability to climate change (35, 38, 50–53).

To counteract some of the negative ecological consequences of agricultural intensification, government programs in the US (e.g., Environmental Quality Incentives Program and Conservation Stewardship Program; 54, 55) have been created to incentivize better management practices (e.g., no-till farming and cover cropping). Landscape diversification through restoration of grassland prairies also has been linked to the enhancement of ecosystem services (e.g., habitat quality, pollination, and water quality and retention) in homogenized croplands (42, 56). For example, the integration of perennial vegetation in the form of “prairie strips” can create ecological buffers that reduce excess runoff of fertilizer and serve as resource islands for wildlife in crop-dominated agroecosystems (42, 57–60). One of the largest US incentive programs, the Conservation Reserve Program (CRP), pays farmers to convert cropland into perennial vegetation with the intention of restoring soil health and creating beneficial wildlife habitat (61). Yet, in some cases, the policies created to derisk instabilities in corn ethanol production (e.g., Renewable Fuel Standard) outcompete the financial incentive of CRP payments (46, 62–64). Therefore, well-intended policies meant to support biofuels may have unintentional negative effects on programs designed to promote conservation in agriculturally dominated landscapes (46). In these instances, income from solar energy could provide the economic relief needed for farmers to pursue conservation strategies that would otherwise be considered financially unattractive.

Unlike extensive corn monocultures, solar facilities can be designed and managed to simultaneously restore perennial vegetation at scale and thus deliver key ecosystem services in agricultural landscapes (13, 17, 19–21, 65, 66). Furthermore, when compared on an energy equivalent basis (conversion to MWh, see *Methods*), solar PV generates the same amount of energy as corn ethanol in just 3.2% of the land-use footprint (Fig. 1*B*; 29). In other words, the energy generated by one hectare of utility-scale solar would require ~31 hectares of corn-ethanol to produce the same amount energy. Potential exists for coupling solar with wind energy, which already occurs at gigawatt scales in the crop-energy matrix of the midwestern US, for example (67), such codevelopment opportunities could improve reliability of intermittent renewable energy systems, mitigate some of the financial risk associated with transmission infrastructure, and further improve land-use efficiency of energy generation in croplands (68–70). Although corn ethanol and solar PV are not a perfect 1-to-1 swap as energy sources (electrons go to the grid and serve several end users while ethanol is primarily used as a fuel additive), electricity demand from the grid is expected to grow 33 to 75% by 2050 (Energy Information Administration) and 33 million electric vehicles are expected to be on the road by 2030 (National Renewable Energy Lab), necessitating a transition to more land-efficient renewable energy generation.

In light of empirical results supporting environmental benefits of ecovoltaic solar in agricultural landscapes, we evaluated how a strategic transition from corn ethanol to solar energy could benefit land-use efficiency of energy production, enhance ecosystem services, and improve landscape diversity in homogenized croplands of the US. We estimated the spatial distribution of land used for corn ethanol production in the US by identifying corn croplands within 160 km (100 miles) of ethanol refineries (71). We then identified corn ethanol croplands within a technically feasible proximity to electrical transmission (≤3.3 km or 2 miles) so as to provide meaningful amounts of electricity (e.g., utility-scale solar energy ≥ 1 MW) to the grid via solar PV. We also identified locations adjacent to wind energy facilities where the coupling of intermittent renewable energy systems could be used to reinforce reliability and potentially lower the cost of interconnection. This allowed us to locate landscapes that could benefit from the strategic integration of ecologically informed solar energy development that enhances ecosystem services and promotes greater landscape diversity in homogenized croplands. We conclude by discussing how an ecovoltaic approach to solar energy development could facilitate broader agricultural diversification that, in turn, supports more resilient agroecosystems under climate change. Our analysis offers a pathway for an energy transition in croplands that facilitates multiple energy and ecosystem service benefits with minimal land-use change via colocation across ag-energy landscapes.

## Results & Discussion

**Fig. 2.**
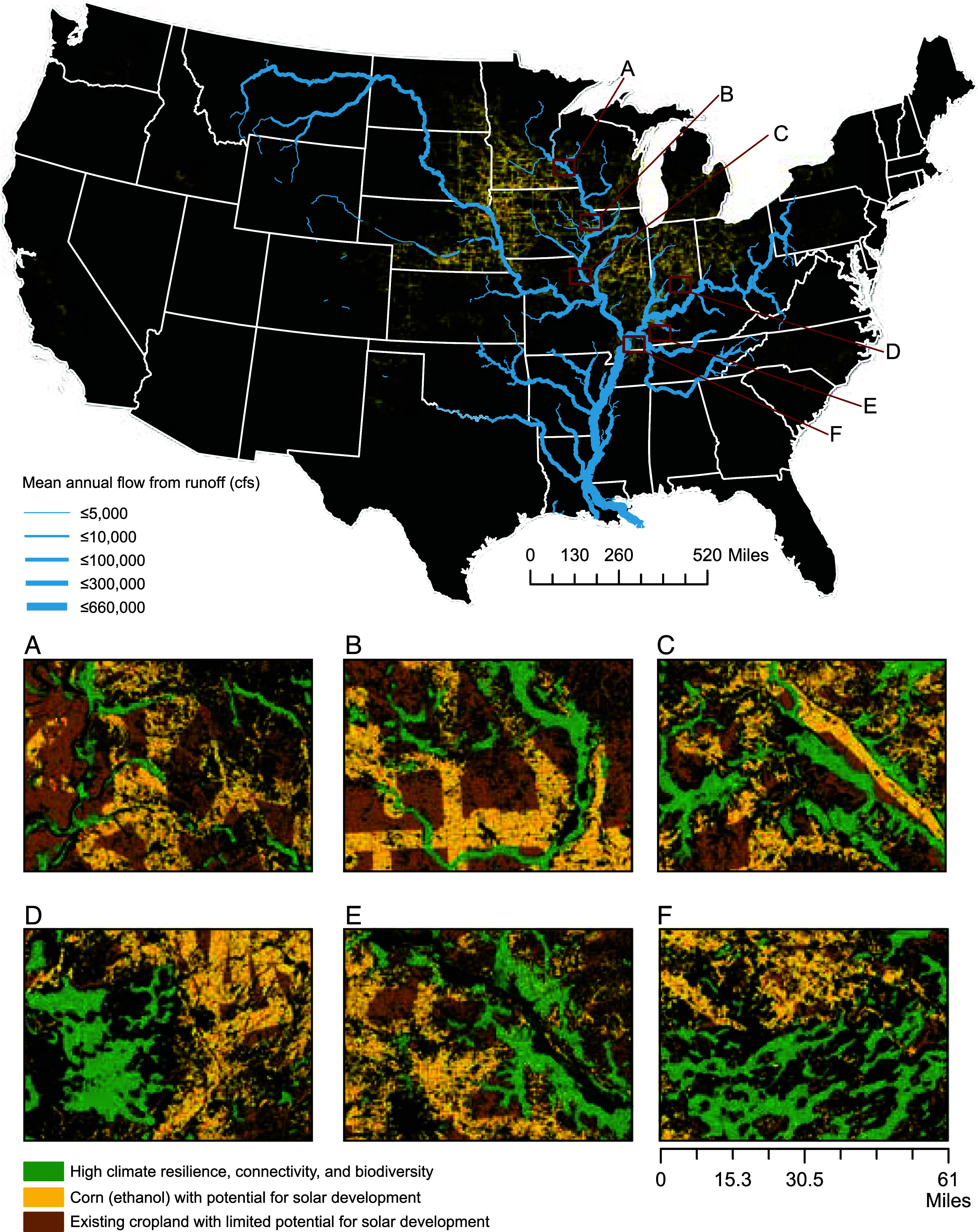
Map of corn ethanol land-cover with high technical potential for solar energy development across the US (yellow) and runoff in the Mississippi River watershed (blue). *Inset* maps (*A*–*F*) depict landscape matrices falling within a subwatershed of the Mississippi River watershed containing either nitrogen or phosphorus transport at the 95th percentile from streams via fertilizer application from agriculture (see ref. 74); matrices include existing agricultural land (brown), potential, ecovoltaic solar energy development with capacity to reduce or eliminate nutrient runoff (yellow), and lands with high climate resilience, connectivity, and biodiversity as defined by The Nature Conservancy’s Resilient and Connected Network (green) (see ref. 75).

We found that a large proportion of corn grown in high frequency (4+ consecutive years) and in close proximity to ethanol refineries is located near existing electrical transmission infrastructure (Fig. 2). These locations, mostly concentrated in the midwestern US, represent immediate opportunities for a transition from corn ethanol to solar energy (Fig. 2 *A*–*F*). At current power densities (*Methods*), this ~391,137 hectares of solar would generate the equivalent amount of energy currently produced by corn ethanol on an annual basis (380,843.5 GWh; Fig. 1*B*), increasing the share of utility-scale solar energy in the US from 3.9 to 13% (72). Furthermore, if 46% of land currently used for corn ethanol were converted to solar, it could generate enough energy (5,340 TWh) to meet 100% of 2050 decarbonization goals for the US (73). We estimate that Illinois, Minnesota, Kansas, Texas, and Nebraska have a combined ~1,200,000 ha where solar energy could replace corn grown for ethanol and simultaneously be colocated with existing wind energy facilities (Table 1), contributing an additional ~23% to national decarbonization goals, meeting about 60% of the expected contribution from solar (2140 TWh, 73).

**Table 1.** Land area in states where corn grown for ethanol is currently colocated with wind energy

State	Corn ethanol + wind (ha)	Contribution to national decarbonization
Iowa	450,188	8.2%
Illinois	220,246	4.0%
Minnesota	204,110	3.7%
Kansas	151,730	2.8%
Texas	148,710	2.7%
Nebraska	101,358	1.9%

These estimates represent spatial extent of potential conversion from corn ethanol to ecovoltaic solar and wind energy colocation. We estimated contribution of this potential land-use conversion as a percentage of 2050 national decarbonization goals (73).

### Enhancing Ecosystem Services and Landscape Diversity.

Many scientists have argued the potential benefits of utilizing solar facilities for habitat enhancement that improves ecosystem services and food and cover for wildlife (9, 13, 17, 19, 65, 66, 76–83). In conventional croplands, additional ecosystem services are often secondary to crop yield (84), and, as such, excessive synthetic fertilizer application has created detrimental feedbacks for surface and ground water (35, 46). Through spatial analysis, we identified multiple locations in the Mississippi River System that disproportionately contribute (95th percentile; 74) to nitrogen and phosphorus transport via farm fertilizer, and where corn is grown in high density with a high likelihood of being processed for ethanol (Fig. 2, panels *A*–*F*). In these locations, solar coupled with perennial vegetation, which acts to stabilize soils, retain sediments, and reduce nutrient runoff by as much as 85% (58, 85), has the potential to meaningfully reduce nutrient leeching into groundwater as well as decrease downstream impacts to river basins and coastal ecosystems that are tightly linked to excessive use of fertilizers (41, 42, 86, 87). Furthermore, our calculations indicate that converting ~391,137 ha of corn (the amount of land needed for solar PV to offset the energy equivalent of corn ethanol) into unfertilized perennial vegetation would reduce nitrogen and phosphorous application by ~54.8 million kg and ~26.3 million kg, respectively (88, 89). Thus, combined actions of fertilizer curtailment via land-use change and mitigation of impacts to larger waterways through vegetation-mediated filtration could both be realized through an ecologically informed approach to solar development in croplands.

Integration of perennial vegetation into monoculture annual cropping systems provides habitat and resource islands, subsequently improving biodiversity of agriculturally and ecologically important plants and animals at the landscape level (41, 59, 60, 90, 91). Further, evidence for the successful establishment of diverse plant communities and subsequent use of floral resources by native pollinator species in solar facilities (20, 21) are encouraging signs for the potential of ecovoltaics to enhance these services while simultaneously producing renewable energy in the midwestern US. PV-altered microclimates have also been shown to extend the growing season and shift the timing of floral resources (92, 93), opening up opportunities for exploring whether solar arrays alter multi-trophic processes through niche differentiation. Future research that explores how PV arrays can act as a potential mitigant for phenological mismatch between flowering plants and the insects that pollinate them will be critical in understanding ecovoltaics propensity for enhancing wildlife (81). More broadly, the benefits of landscape diversification have been shown to improve agricultural yields, stability, and resilience in highly homogenized croplands (94–98). Synergies between solar energy development and sustainable agroecosystems may be found as research findings accumulate.

### Realizing a Transition to Solar in Croplands.

A key tenet of ecovoltaics is the avoidance of solar development in pristine and sensitive native ecosystems (13). To this point, scientists have long argued that there are more than enough degraded and abandoned lands available to meet even the most optimistic renewable energy goals (9, 13, 99, 100). However, several factors, including transmission capacity, geographical remoteness, and economies of scale (101, 102), currently limit our ability to constrain solar development to degraded landscapes (e.g., superfund sites, abandoned farmland) and the built environment (e.g., rooftops and parking lots). Although installing solar energy facilities in croplands may seem paradoxical with respect to sustainable land management [e.g., construction practices like grading and removal of vegetation can be detrimental to ecosystem services (16)], our findings suggest that an ecovoltaic approach that promotes ecosystem service enhancement in ecologically marginalized agroecosystems has potential to facilitate land-use synergies for a more sustainable future.

Given corn ethanol production and consumption trends in the US (27), we estimate that converting 3.2% of corn-ethanol land into solar would have little effect on domestic biofuel markets. Thus, the gradual diversification of the energy matrix could occur within a time frame in which land-energy management strategies can be effectively codeveloped and evaluated. Market subsidies that support ethanol production could then be reappropriated to concurrently support other forms of renewable energy that promote multifunctional landscapes, benefit ecosystem health (e.g., soil stabilization, water filtration, carbon sequestration), and serve as a catalyst for cropland diversification while accounting for agricultural socioeconomics in agrarian communities. Diverse forms of energy generation may be needed to realize sustainable land use in the energy-agriculture matrix, and future research that holistically evaluates the social benefits of ecosystem services could be useful. Even without subsidization, solar energy generation has potential to alleviate economic burdens that act as a barrier to cropland diversification (103). Although solar leases and crop prices vary drastically, some estimates report that landowners who lease croplands to solar developers earn 3 to 4× more per acre than most crops at current market prices (104).

A more sustainable and resilient food system requires efficient use of croplands, and in the US, our results indicate that a transition from corn ethanol to ecovoltaic solar has great potential to act as a catalyst for more sustainable agroecosystems overall. Enhanced pollinator services could create opportunities for cultivation of more diverse crops (105). In turn, landscape diversification could improve climate resilience (e.g., mitigation of extreme weather conditions on yield, protection from novel pests and pathogens; 95, 99, 106–108), particularly in the midwestern US where a large proportion of agricultural land is cultivated as vast monocultures. Ecovoltaic solar, in tandem with precision use of fertilizers and irrigation, cover cropping, low and no-tilling, and other management practices that focus on enhancement of ecosystem services (109, 110), provides a potential pathway for more sustainable agroecosystems overall.

As the transition to renewable energy proliferates in croplands, ecological tradeoffs, and synergies can be evaluated to envision sustainable land management in agricultural systems. Restoring functional, perennial plant-dominated ecosystems within utility scale solar facilities has been achieved at scale in the midwestern US with existing technologies, thus it offers a realistic solution for an informed energy transition that simultaneously promotes ecosystem health. Additionally, potential opportunities for sustainable energy transitions regionally can be extrapolated to envision similar strategic land-use change globally. In our analysis, we identified several potential land-use and ecosystem service benefits that could be realized by converting a small proportion of corn ethanol croplands into ecovoltaic solar arrays. With more solar energy development on the horizon, our analysis offers an evidence-based pathway for successful, colocated energy landscapes that can improve the relationship between energy development and ecosystems.

## Methods

### Corn Land-Cover Frequency.

We identified corn land-cover frequency from a time-series analysis of 30-m resolution US Department of Agriculture Cropland Data Layer (CDL) (24) dataset for all available years (2008 to 2023) for the continental US (CONUS). We obtained a spatial representation of corn land-cover frequencies across CONUS (*SI Appendix*, S4), by following general recommendations for processing and analyzing CDL data outlined in Lark et al. (111) and Lark et al. (33). First, we reclassified all data from the CDL into two broad categories—corn and noncorn—for each year (i.e., 2008 to 2023) to facilitate computing power and detection of broad agricultural land use across CONUS. We defined spatial identification of corn (CDL class 1), double crop winter wheat/corn (CDL class 225), double crop oats/corn (CDL class 226), double crop triticale/corn (CDL class 228), double crop barley/corn (CDL class 237), and double crop soybeans/corn (CDL class 241), which cover all possible situations where corn could be grown for ethanol production. Having generated a binary raster for each year of available data, we merged each raster into a stack, resulting in a single raster dataset describing the frequency of corn land-cover across years in CONUS. To this dataset of corn land-cover frequencies, we applied a majority filter with a spatial connectivity of eight neighbors and a replacement threshold of the majority of spatially contiguous pixels (30 m resolution) to enhance the classification of corn frequencies and smooth out any potential anomalies (112). Following this reclassification and filtering process, resultant data represented agricultural fields that have grown corn for four or more consecutive years. We then applied a minimum mapping unit of 2 ha to aid in overall classification and accuracy of the resultant dataset (see ref. 111). We reclassified patches less than 2 ha as no data thus removing them from consideration in the analysis. This general approach for determining corn land-cover frequencies of 2 ha or greater across CONUS leverages the high accuracy of the CDL data (>98% for all years) and provides a robust and proven methodology for land-use analysis of agriculture landscapes, using publicly available geospatial datasets (111).

### Relating Corn Ethanol and Potential Solar Energy Development.

Having determined the spatial distribution of corn land-use frequency in CONUS, we performed a spatial analysis of corn grown for ethanol production that met the following criteria: 1) corn land use within 100 miles from an ethanol refinery; and 2) consistent corn land-use (4+ consecutive years). We obtained locations of ethanol refineries from the US Energy Information Administration (*SI Appendix*, S5; 28); these locations are accompanied by information related to production capacity (million gallons per year) (28). We set a maximum buffer distance for corn land-cover frequency of 160 km (100 miles) from an ethanol refinery (71); we assumed corn grown outside of 160 km of an ethanol refinery to follow a different end-use path (e.g., animal feed, food and beverage, or industrial uses; *SI Appendix*, figure S1, 113). Given the known value of agricultural lands as recipient environments for solar energy development (i.e., flat and thereby feasible to develop; great solar resources), interconnection can be a primary driver of technical potential for solar energy development in croplands. We overlaid a 3.2 km buffer around transmission lines, which serves as an interconnection threshold for potential utility-scale solar facilities (114). By extracting corn land-use frequencies that fall within both the 160 km buffer around ethanol plants and the 3.2 km buffer around transmission lines, we generated a dataset representing areas where corn is consistently grown for ethanol production that also meet proximity criteria for suitability analysis of solar energy infrastructure.

### Land-Use Efficiency of Solar Energy Production as a Corn Ethanol Replacement.

We estimated the land area required for utility-scale solar energy (≥1 MW; 114) to match and replace electricity generation potential of corn ethanol in 2022 by leveraging multiple datasets related to corn and ethanol production and utility-scale solar energy. In 2022, the US produced 15,360,696,000 gallons of corn-derived ethanol (27); of the 13,722,442,966 bushels of corn grown in the US in 2022, 5,175,000,000 bushels were processed for ethanol (29, 113) at a rate of approximately 2.9 bushels of corn per gallon of ethanol. Using a 2022 average yield rate of 429.7 bushels per ha for the top 10 ethanol producing states in the US (29), we estimated 12,268,389 ha (30,315,849 acres) dedicated to corn ethanol, around 38% of corn harvested in the US in 2022 (Fig. 1*B*). To determine the energy content of ethanol production, we converted gallons of ethanol into British thermal units (Btu) based on conversion factors published by the US EIA for ethanol; the 2022 estimate is 84,595 Btu per gallon (115). Therefore, total energy content of ethanol in 2022 equated to ~1.299 quadrillion Btus, which is equivalent to 380,843,516 MWh of electricity, assuming 3,412 Btu per kilowatt hour (115). To estimate the land area of utility-scale solar energy necessary to generate 380.8 million MWh of electricity, we first converted generation into capacity based on Eq. **1**. Using an average capacity factor for utility-scale solar energy of 24.7% (116), we estimate a total solar energy capacity of 176,013 MW. Using a median power density of 0.45 MW_ac_ per hectare (117), the land requirement for 176,013 MW is approximately 391,137 hectares (Fig. 1*B*, 117) and represents the total area required to offset the energy potential of ethanol production in 2022.

[1] $${\text{Capacity}}_{\text{MW}}=\frac{{\text{Generation}}_{\text{MWh}}}{\left(\mathrm{hours}\;\mathrm{per}\;{\mathrm{year}}^{*}\mathrm{capacity}\;\mathrm{factor}\right)}.$$

## Supplementary Material

Appendix 01 (PDF)

## Data Availability

Data used in this study are cited in the main text and supplemental materials and are described in detail in the *Methods*. Code developed for and used in this study are available in the following GitHub repository (https://github.com/GallaherAdam/Ecovoltaics-CONUS) (118). A citable Zenodo repository, DOI: 10.5281/zenodo.15125777, has also been added to ensure accurate code attribution (119).
