# Supplementary material for: Ecologically informed solar enables a sustainable energy transition in US croplands
Source: Proc Natl Acad Sci U S A. 2025 Apr 21;122(17):e2501605122. doi: 10.1073/pnas.2501605122 (PMC12054829; doi:10.1073/pnas.2501605122)
Supplement: Supplementary file 1 — Appendix 01 (PDF) [file pnas.2501605122.sapp.pdf]

Supplemental Information

Title: **Ecologically informed solar enables a sustainable energy transition in U.S. croplands**

Authors: Matthew A. Sturchio[1,2], Adam Gallaher[1], Steven M. Grodsky[3]

[1]*Department of Natural Resources and the Environment, Cornell University, Ithaca, NY, USA*

[2]*Department of Biology, Colorado State University, Fort Collins, CO, USA*

[3]*U.S. Geological Survey, New York Cooperative Fish and Wildlife Research Unit, Department of Natural Resources and the Environment, Cornell University*

*Correspondence: mas896@cornell.edu

Any use of trade, firm, or product names is for descriptive purposes only and does not imply endorsement by the U.S. Government.

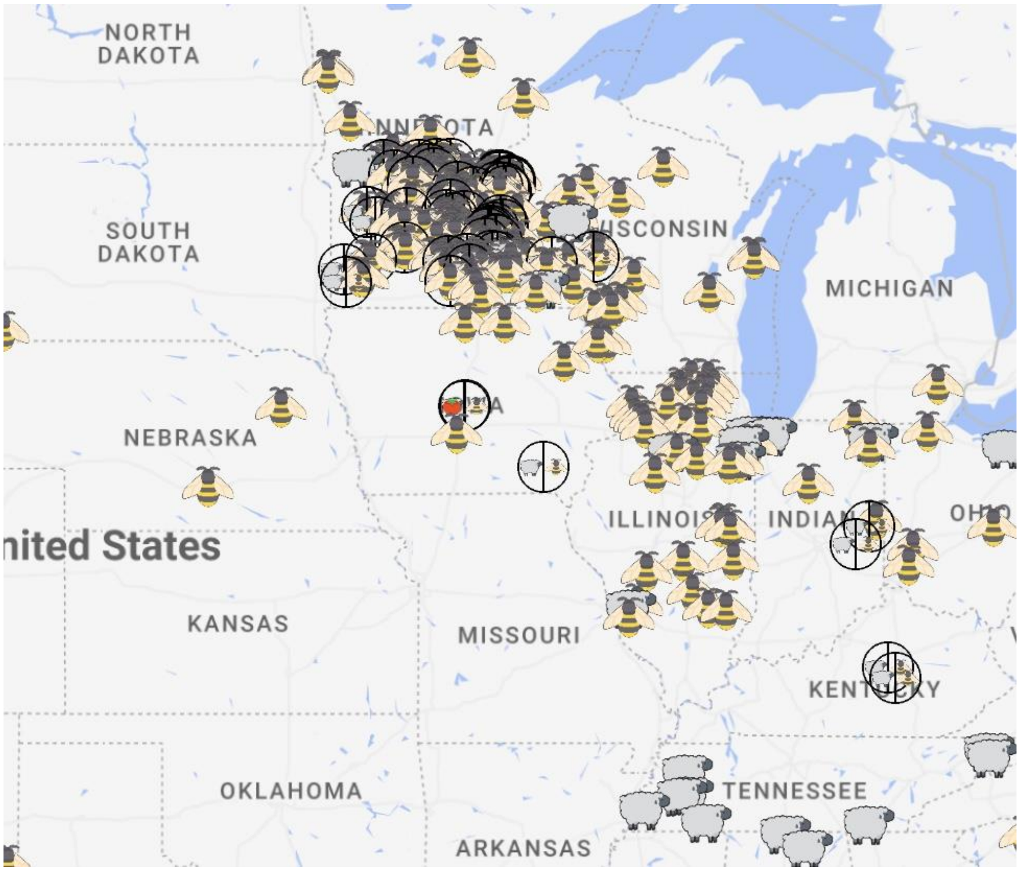

S1. Map of solar arrays managed with co-located perennial vegetation throughout the Midwestern United States (Source: https://openei.org/wiki/InSPIRE/Agrivoltaics_Map). Accessed October 28th 2024.

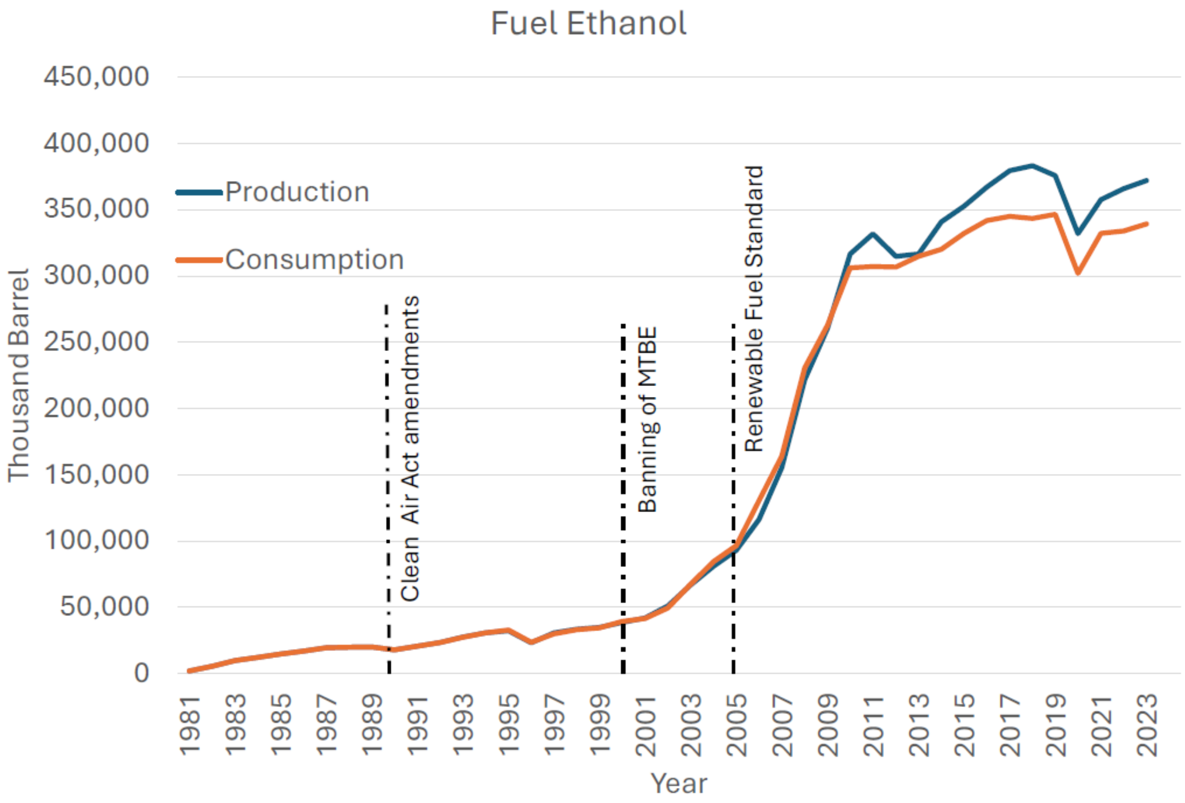

S2. Production and consumption of ethanol in the United States from 1981 to 2023[1].

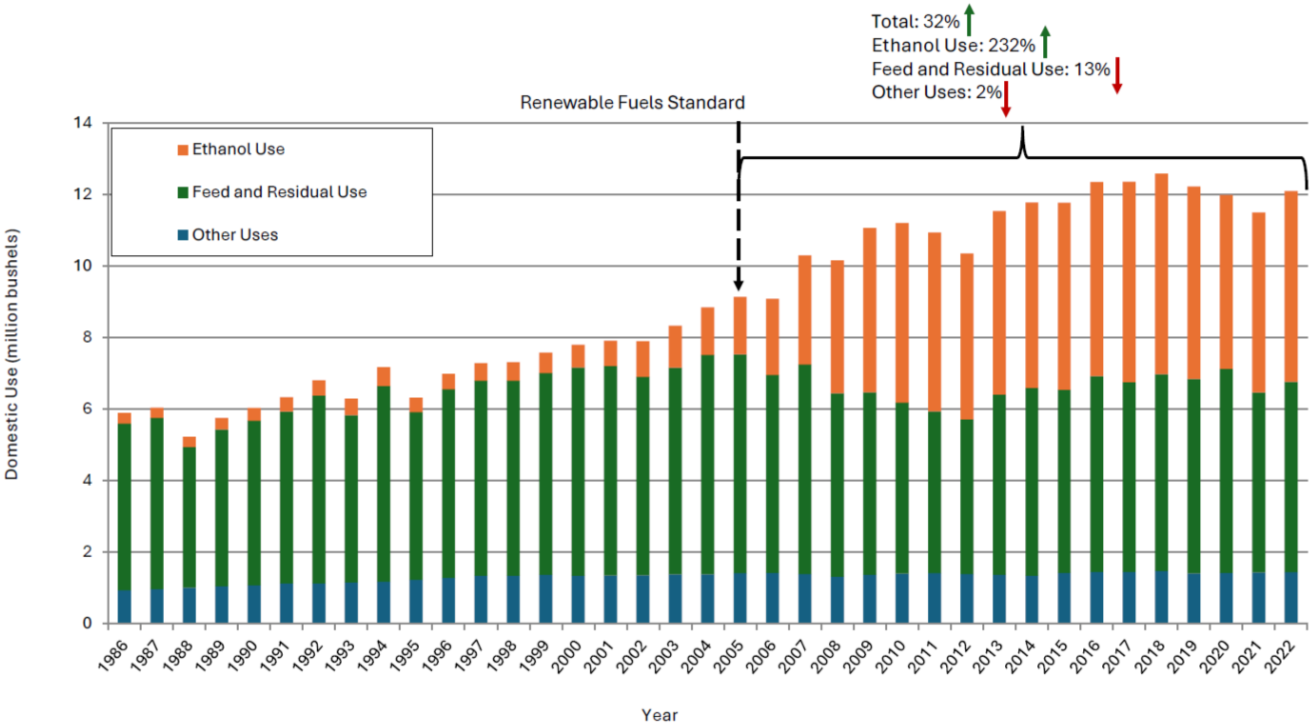

S3. End use of corn in the United States: 1986 to 2022[2].

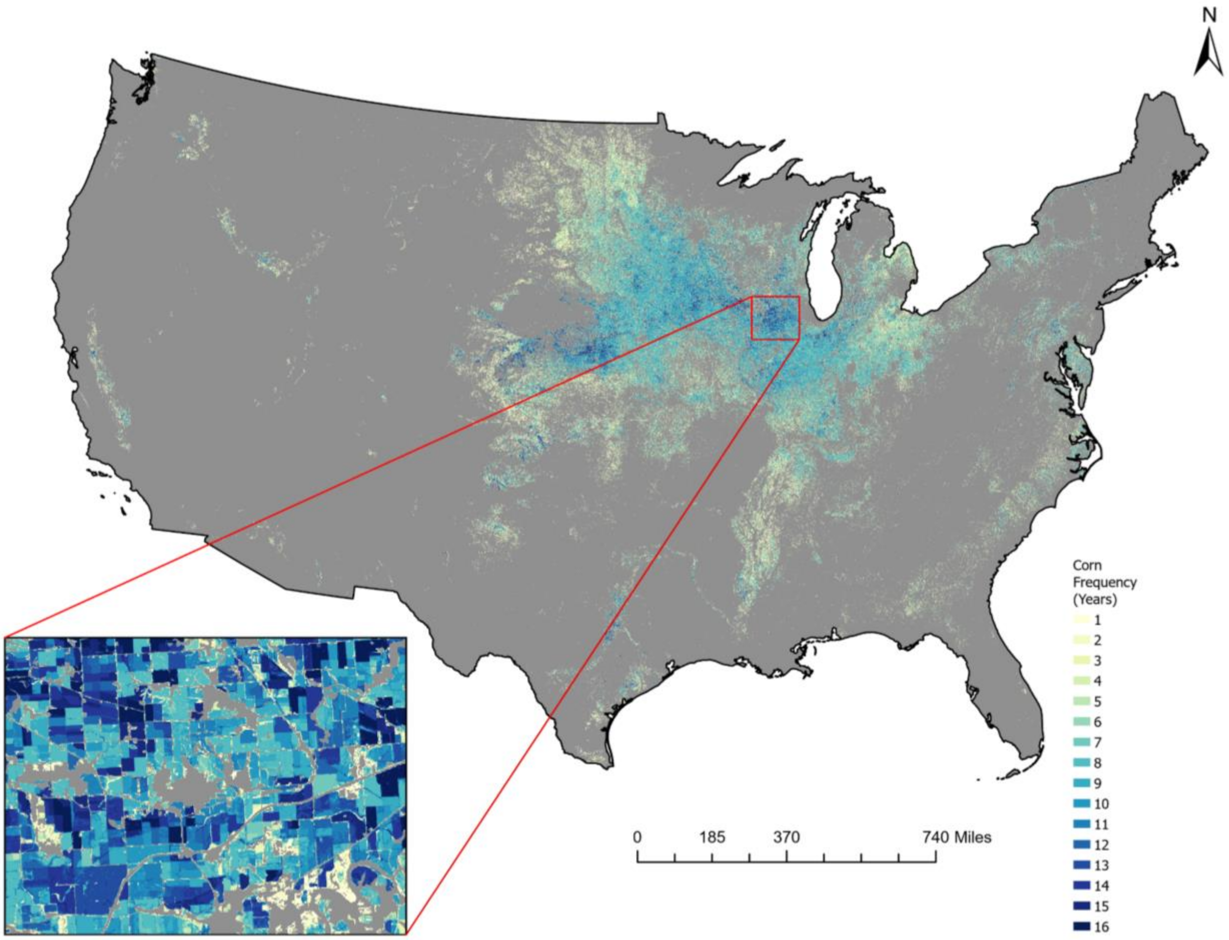

S4. Corn land-use frequency for CONUS from 2008 to 2023[3].

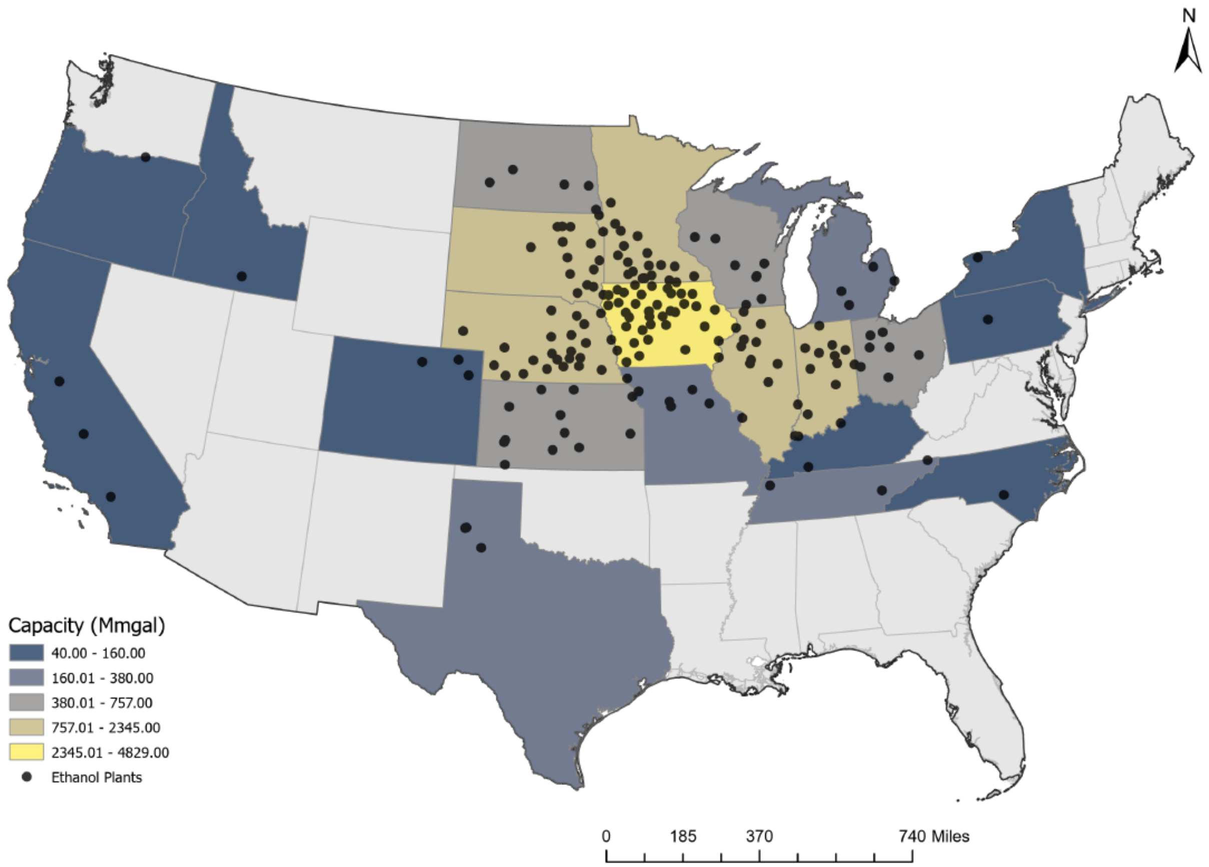

S5. Production capacity of ethanol refineries by state for the contiguous United States. Black points represent the geographic location of ethanol refinieries[4].

Supplemental References

1. U.S. Energy Information Administration (2024a), Total Energy Data Browser, accessed August 16, 2024, at https://www.eia.gov/totalenergy/data/browser/?tbl=T10.03#/?f=A&start=1981&end=2023&charted=8-19

2. U.S. Department of Agriculture, Economic Research Service. Feed Grains Database. Accessed October 13, 2024, at https://www.ers.usda.gov/data-products/feed-grains-database/feed-grains-yearbook-tables/

3. U.S. Department of Agriculture, (2023) National Agricultural Statistics Service 20240131, Cropland Data Layer. Data available at https://croplandcros.scinet.usda.gov

4. U.S. Energy Information Administration (2024b), Ethanol Plants, accessed August, 15, 2024, at https://atlas.eia.gov/datasets/eia::ethanol-plants-1/about
